# Supplementary material for: TmSpz4 Plays an Important Role in Regulating the Production of Antimicrobial Peptides in Response to Escherichia coli and Candida albicans Infections
Source: Int J Mol Sci. 2020 Mar 9;21(5):1878. doi: 10.3390/ijms21051878 (PMC7084639; doi:10.3390/ijms21051878)
Supplement: Supplementary file 1 [file ijms-21-01878-s001.zip › Supplementary Figure 1.docx]

>TmSpz4

AGAGCATCTGACAGTGGAAACCTCATTTCTCGAGTAAACAGGGGTCCGCGAGTCCAGTTC

ACCGGTACCACTCCGTCGGTCAAGACGCTGGTCCGCTCCGAGAAAACGCGAAGTACCATG

CGGATCCACCTCGCCCAGTGCTGTGCCCATCGCTGGTCCACGCTCTGCGCCTGAACGAC**G**

**>**

**ACGGTGCCCACGAACTAT**GCGAC

**>>>>>>>>>>>>>>>>>> TmSPZ4-cloning-FullORF-Fw**

ATGTTTCGCCTAATCCTGCTCCTCTTATACGCCCGGCGAAGCCTCGCCTACGGTTTCGAC

TCCGGGGGCGACCAAGGCGCCTGCGGCAGGCAGTACCGCGCCGGGAAGATGCACCGCCCC

CCGTGCGATCTCTCCGCGGGGTACTGCACTTCAGCCGGGGCGGCATACCCGTGGCACGCC

GTCAAGAGGTTCGTCAGAGAGAACCACGGCCTGATGCGGAGGATGTACGGCGACCAGCGC

CACGGCCAGGTCCTCAAGGCTGAGCTGGAGGACGTGGGGTTGCAACCCCTCGACCACCAG

CAGCGATTCGACGACCGCAAAATGTACGGCAAGCAAGAGTACCATTTCAGCACCGACAAC

GAGCTGGGCCGCGACAGCGAACCTAGGTTCTTGAGGGAGGACGACATCAGCAACGATGAC

GTCCTCAACACCAAGTTCGTCTACTCGGAGGAGCCCTCCGTCAGCATCAAGCTGGCGGAC

CTGAGGACCGCGCCTCACTTCAGACCCACGACCCACAAGTCCACGACGGAAGAACCGCCG

AAGACCACCACCGAAGAGGCGACGACTCCGACGGAGACCACTACGGCGGAGTCCACCACC

>>>

GACACAACCACCAAAAGCGACAGCGCGAGCCCCACGGAAGCCGGCGCGACGAGCGAAGGC

>>>>>>>>>>>>>>>>>**TmSPZ4-cloning-Fw**

GAGAGTCCGGGGGCGATGCTCTTCCAGGACGCGGAGCAGAGCAAGGACGCCCACAAGGTC

>>>>>>>>>>>>>>>>>>>**TmSPZ4-qPCR-Fw**

CAAGTCGACTACCAAATGAAAGGAGTGAACGCGTGTCCGGTGAAGGAGGAAGTGGTGGCG

**TmSPZ4-qPCR-Rv**<<<<<<<<<<<<<<<<<<<<<<

CCGTTCTGGGCCAACAACACCCGCGGCGAGGTCCTCGCACTGCTCAACATGTACCCCTTC

GAGCAGTACGTCCACTGGGAGAAGTGCACTCACGAGCACCGCCAGATGTACTGTCGCGAT

>>>>>>>>>>>>>>>>>>>

GGATGCCGCTGCGAGCAACAGTATCGCCTACACAGACTGTTGGCCTACGACCCCAACAAC

>**TmSPZ4-dsRNA-Fw**

GAGTGTCGAGGCATCTTTTCCGACTGGTTCAGGTTTCCGTCGTGTTGCGTCTGCAAGTGCTACAGCATCCCTCCGGCCGAATTTAGGGTCACGTCGAGGAGTCCACGAGCTTACAGCGACCAAGAGGTGCCCGACTGGTACAGAAGGAAAAGTGAAGCGATTTTTTCCGGAGATCACGGATGA

<<<<<<<<<<<<<<<<<<<< <<<<<<<<<<<<<<<<<<<<

**TmSPZ4-dsRNA-Rv** **TmSPZ4-cloning-Rv**

AGGTCCATAAGTGCCTCAAGCGAAAC**ATGTCAAGGCTCGTGCTCTT**TTATTTTACGGACA

**TmSPZ4-cloning-FullORF-Rv** **<<<<<<<<<<<<<<<<<<<<**

TTTTGTAACGCATTCTGTACATTTATACATTATTCACATTAACATTTGTGTAGATATTCA

TACTAAAAGGAATTAAACATTGTTGTGTTCTGACAGCTGTTTCGTCATTTTCTATTCCAA

ATAGTGTACGCCATTTTCTGATGAAGAAAAATTGCTTTGTTTTCAACATGATTTTAAATA

TTTTGTTAATGTAAAGTAAAATATTTGTATTGTTATCCAACGCTTCGGTCAGTCTGACCG

ATTCTAATTACCTAATCGATTTTAAAATTTAAACTGTTAGAAAAACAGTATATCCAAGCA

TATCAGGACCATATTGTCTTTTTTGACAAACTTGTCAGTCTTGTCGTTGTCACTTCCTAA

TTGTTCCGTCAGATGTTTAGTGATTTTACCGTGGTGAGTTTGGTAGAAAACTCATGTGGA

AAAATCGAAATAAAAGCCACCATTAAACTGATTT

MFRLILLLLYARRSLAYGFDSGGDQGACGRQYRAGKMHRPPCDLSAGYCTSAGAAYPWHA

VKRFVRENHGLMRRMYGDQRHGQVLKAELEDVGLQPLDHQQRFDDRKMYGKQEYHFSTDN

ELGRDSEPRFLREDDISNDDVLNTKFVYSEEPSVSIKLADLRTAPHFRPTTHKSTTEEPP

KTTTEEATTPTETTTAESTTDTTTKSDSASPTEAGATSEGESPGAMLFQDAEQSKDAHKV

QVDYQMKGVNACPVKEEVVAPFWANNTRGEVLALLNMYPFEQYVHWEKCTHEHRQMYCRD

GCRCEQQYRLHRLLAYDPNNECRGIFSDWFRFPSCCVCKCYSIPPAEFRVTSRSPRAYSD

QEVPDWYRRKSEAIFSGDHG*

**Supplementary Figure 1**. The full length cDNA sequence of *TmSpz4*. The symbol “>” (forward) and “<” (reverse) indicates the position of primers used for real-time analysis and dsRNA synthesis.
