# Supplementary material for: TmSpz4 Plays an Important Role in Regulating the Production of Antimicrobial Peptides in Response to Escherichia coli and Candida albicans Infections
Source: Int J Mol Sci. 2020 Mar 9;21(5):1878. doi: 10.3390/ijms21051878 (PMC7084639; doi:10.3390/ijms21051878)
Supplement: Supplementary file 1 [file ijms-21-01878-s001.zip › Supplementary Figure 2.docx]

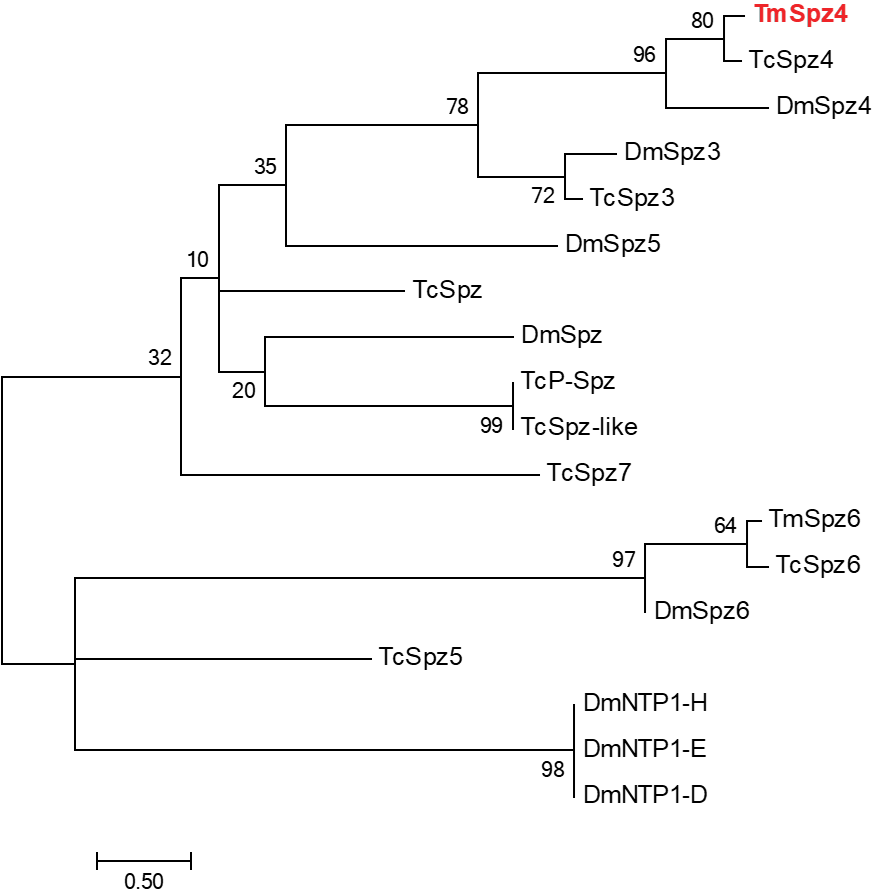


**Figure 2.** The molecular phylogenetic analysis of insect Spz4 homologs protein.

Phylogenetic analyses of *Tm*Spz4 homologues protein were performed using the Clustal X2 and the phylogenic tree was constructed by MEGA7 programs using the maximum likelihood and bootstrapped of 1,000 replications. The following Protein sequences were used to construct the phylogenetic tree. *Dm*Spz4; *Drosophila melanogaster spatzle 4* (AAF53100.2), *Dm*Spz6; *Drosophila melanogaster* spatzle 6 (AAF47261.1), *Dm*Spz5; *Drosophila melanogaster* spatzle 5 (AAF47694.1), *Dm*Spz; *Drosophila melanogaster* spatzle (AAF82745.1), *Dm*Spz3; *Drosophila melanogaster* spatzle 3 (AAF52574.2), *Dm*NTP1-H; *Drosophila melanogaster* neurotrophin 1, isoform H (AGB94113.1), *Dm*NTP1-E; *Drosophila melanogaster* neurotrophin 1, isoform E (ACZ94621.1), *Dm*NTP1-D; *Drosophila melanogaster* neurotrophin 1, isoform D (NP_001163348.1), *Tc*Spz7; *Tribolium castaneum* spatzle 7 (EEZ99267.2), *Tc*Spz4; *Tribolium castaneum* spatzle 4 (EFA09263.2), *Tc*Spz5; *Tribolium castaneum* spatzle 5 (EEZ97725.1), *Tc*Spz3; *Tribolium castaneum* spaetzle 3 (NP_001153625.1), *Tc*Spz6; *Tribolium castaneum* spatzle 6 precursor (NP_001164082.1), *Tc*Spz; *Tribolium castaneum* spaetzle (EEZ99207.1), *Tc*P-Spz; *Tribolium castaneum* PREDICTED: protein spaetzle (XP_008201191.1), *Tc*Spz-like; *Tribolium castaneum* spaetzle-like protein (EEZ99268.2).
